# Effects of the COVID-19 Pandemic and Post-Pandemic Changes on the Diagnosis, Treatment, and Mortality of Hepatocellular Carcinoma in a Tertiary Center in Western Romania

**DOI:** 10.3390/cancers17101660

**Published:** 2025-05-14

**Authors:** Calin Burciu, Bogdan Miutescu, Renata Bende, Deiana Burciu, Tudor Voicu Moga, Alina Popescu, Alexandru Popa, Felix Bende, Eyad Gadour, Adrian Burdan, Dana Iovanescu, Mirela Danila, Roxana Sirli

**Affiliations:** 1 Department of Gastroenterology, Faculty of Medicine, Pharmacy and Dental Medicine, “Vasile Goldis” West University of Arad, 310414 Arad, Romania; calin.burciu@umft.ro (C.B.); danagastro@yahoo.com (D.I.); 2 Center for Advanced Research in Gastroenterology and Hepatology, “Victor Babes” University of Medicine and Pharmacy, 300041 Timisoara, Romania; bende.renata@umft.ro (R.B.); deiana.vuletici@umft.ro (D.B.); moga.tudor@umft.ro (T.V.M.); popescu.alina@umft.ro (A.P.); popa.alexandru@umft.ro (A.P.); bende.felix@umft.ro (F.B.); ghita-adrian.burdan@umft.ro (A.B.); danila.mirela@umft.ro (M.D.); sirli.roxana@umft.ro (R.S.); 3 Department of Gastroenterology and Hepatology, “Victor Babes” University of Medicine and Pharmacy Timisoara, 300041 Timisoara, Romania; 4 Department of Gastroenterology and Hepatology, “Pius Brinzeu” County Emergency Clinical Hospital, 300723 Timisoara, Romania; 5 Multi-Organ Transplant Centre of Excellence, Liver Transplantation Unit, King Fahad Specialist Hospital, Dammam 32253, Saudi Arabia; eyadgadour@doctors.org.uk; 6 Department of Medicine, Faculty of Medicine, Zamzam University College, Khartoum 11113, Sudan

**Keywords:** hepatocellular carcinoma, COVID-19, survival

## Abstract

Background/Objectives: Access to healthcare services was significantly restricted during the COVID-19 pandemic, leading to changes in the management of hepatocellular carcinoma (HCC). However, limited research has examined how these changes evolved post-pandemic. This study evaluated the impact of the pandemic at a tertiary center in Romania, focusing on diagnosis rates, treatments, and survival outcomes. Methods: A retrospective study conducted at Timișoara County Hospital divided patients into three equal cohorts of 23 months each: the pre-pandemic period (PreP: 1 May 2018–31 March 2020), the pandemic period (PandP: 1 April 2020–28 February 2022), and the post-pandemic period (PostP: 1 March 2022–31 January 2024). Newly diagnosed HCC cases were evaluated for the tumor stage, biological markers, and treatment received during each period. A survival census was conducted nine months after the diagnosis. Results: During the PandP and PostP periods, the numbers of newly diagnosed HCC cases decreased to 58 cases (*p* < 0.001) and 64 cases (*p* < 0.005), respectively, representing reductions of 38.3% and 31.9% compared to the PreP period, which had 94 cases. The proportion of patients in the BCLC-B stage increased from 31.9% in the PreP period to 50% during the PandP period (*p* = 0.0401), with fewer BCLC-A-0 cases (17% vs 5.1%; *p* = 0.059) during PandP. The tumor characteristics, BCLC classification, and TNM staging showed no significant differences between the PreP and PostP periods. Systemic therapy was the most commonly used treatment (39.7–50%). No significant differences were observed across treatment types when comparing all three periods (*p* > 0.05). The median follow-up times in the PreP, PandP, and PostP periods were 157.5, 159.5, and 183.5 days, respectively, with no statistically significant differences. The survival curve showed no statistically significant differences in survival between the groups at the nine-month follow-up (*p* > 0.05). Conclusions: The COVID-19 pandemic decreased HCC diagnoses, with only a partial rebound in the PostP period that did not reach PreP levels. While the PandP period showed worsening BCLC staging and an increase in tumor numbers, the tumor stage and treatment in the PostP period were similar to those in the PreP period. Similarly, the nine-month survival rates remained similar across all three periods.

## Simple Summary

The COVID-19 pandemic disrupted healthcare services worldwide, making it more difficult for patients with Hepatocellular Carcinoma (HCC) to receive a timely diagnosis and treatment. This study focused on understanding how the pandemic affected the management of HCC in Romania, comparing the periods before, during, and after the pandemic. The goal was to identify changes in diagnosis rates, treatment approaches, and survival outcomes over time. The findings highlight an important decrease in HCC diagnoses during the pandemic, with only a partial recovery afterward. Moreover, the stage of HCC in the pandemic period worsened, with more BCLC-B on account of BCLC-A. However, the types of treatment and nine-month survival rates remained consistent across all periods. This study aimed to provide an understanding of how healthcare systems were affected by restrictions associated with the pandemic and the need for future exploration of the consequences in the post-pandemic period.

## 1. Introduction

In March 2020, the World Health Organization (WHO) officially declared COVID-19 a global pandemic [1]. This declaration marked a significant shift in healthcare priorities, as medical resources were redirected to focus on treating COVID-19 patients. Simultaneously, social distancing rules and restrictions limited people’s access to medical services [2]. Compounding this challenge was a widespread fear of contracting the SARS-CoV-2 virus, discouraging many from seeking care at hospitals [3]. Although telemedicine solutions were introduced to mitigate the pandemic’s impact on healthcare, these systems often did not include screening for hepatocellular carcinoma (HCC) [4].

Liver cancer is a major global health issue, accounting for 8.3% of all cancer-related deaths and ranking as the third leading cause of cancer mortality [5]. Among liver cancers, HCC is the most common type, comprising 80% of cases in this category [6]. Unfortunately, the number of HCC cases is expected to grow in the coming years [7]. Over 90% of these cases occur in patients with chronic liver disease, with advanced fibrosis or cirrhosis being the leading risk factor. In patients with cirrhosis, the annual risk of developing HCC ranges between 1% and 6% [8].

As with other types of cancer, early detection plays a critical role in improving the treatment outcomes and survival rates for patients with HCC [9]. The European Association for the Study of the Liver (EASL) guidelines recommend screening for at-risk patients through biannual abdominal ultrasound examinations and biomarkers such as alpha-fetoprotein (AFP) [10]. Screening offers substantial benefits, including the potential for curative treatments and extended survival [11]. However, it is concerning that HCC screening continues to be underutilized, highlighting the need for increased awareness and action [12].

In Romania, HCC presents a significant public health challenge due to its high incidence and mortality rates, ranking second in Europe. The age-standardized rates (ASRs) per 100,000 people are 8.9 for incidence and 8.2 for mortality [13]. Despite these concerning figures, Romania does not have a well-established HCC screening program [14], a gap that the adverse effects of the COVID-19 pandemic on HCC management have likely worsened.

The pandemic caused a decline in screening among high-risk patients, leading to more extended screening intervals [4,14]. In the United States, the screening rates dropped by 44% [15]. As a result, the number of HCC cases diagnosed during the pandemic fell significantly, with early reports indicating reductions of 35–37% [16,17]. These findings were later supported by nationwide studies from Japan [18] and the United States, where analyses of two national databases showed a 12.2% decrease in diagnosed cases [19]. Along with fewer diagnoses, some studies noted that patients were found to have larger tumors at diagnosis [16,20]. However, research involving larger patient populations did not reveal consistent changes in tumor size [21], the proportion of patients with early-stage tumors [22], or Barcelona Clinic Liver Cancer (BCLC) staging between the pre-pandemic and pandemic periods [20].

The pandemic also affected access to treatment for HCC patients, with delays in imaging frequently noted during tumor board discussions [23]. However, no significant decline in the receipt of curative treatments was observed in the United States [22], and similar findings were reported in Japan, where no treatment delays were identified [21]. The mortality and short-term survival rates for HCC patients showed no significant differences between the pandemic and pre-pandemic periods [19]. These results should be interpreted cautiously, as pandemic-related restrictions, healthcare systems, screening programs, and health policies vary across regions. Many studies highlight the need for further research to evaluate the pandemic’s long-term effects on HCC diagnosis, treatment, and survival [19,20,22].

This study aimed to assess the impact of the COVID-19 pandemic on HCC management at a tertiary center in Romania. It compared the pre-pandemic (PreP), pandemic (PandP), and post-pandemic (PostP) periods by analyzing trends in the number of diagnosed cases, treatments received, and survival rates.

## 2. Materials and Methods

### 2.1. Patients and Variables

This retrospective study was conducted at Timișoara County Emergency Clinical Hospital in Romania from 1 May 2018 to 31 January 2024, and included all patients newly diagnosed with HCC. As the largest hospital and referral center in western Romania, as well as a key institution affiliated with the region’s leading medical university, our hospital provided a strong foundation for this research.

To meet the study’s objectives, newly diagnosed HCC patients were divided into three periods. The first period, PreP, spanned from 1 May 2018 to 31 March 2020, before Romania implemented hospital admissions and social distancing restrictions in late March 2020. The second period, PandP, covered 1 April 2020 to 28 February 2022. This period was characterized by limitations such as reduced hospital access, fewer hospitalizations, and restricted availability of hospital beds due to social distancing measures. The third period, PostP, ranged from 1 March 2022 to 31 January 2024, during which all restrictions on patient access to healthcare services were lifted.

Each period was set to 23 months to ensure balanced comparisons and account for the duration of the restrictions. The exclusion criteria included patients diagnosed before the study period and those already undergoing treatment.

Data were extracted from the patients’ medical records to obtain information on the demographic and clinical characteristics, presence of underlying liver disease, tumor staging, and treatment received. The treatment recommendations and decisions were guided by the BCLC guidelines [24], incorporating individualized discussions within the oncology committee for borderline cases while also considering patient preferences. A nine-month follow-up after diagnosis was conducted to evaluate differences in survival across the three time periods.

The study was approved by the Ethics Committee of Timișoara County Hospital (Reference No. 494/17.10.2024) and was conducted in accordance with the principles of the Declaration of Helsinki.

### 2.2. Diagnosis of HCC and Staging

The diagnosis of HCC was made using contrast imaging techniques such as contrast-enhanced ultrasound (CEUS), contrast-enhanced computed tomography (CE-CT), and contrast-enhanced magnetic resonance imaging (CE-MRI). The key imaging features of HCC included hypervascularity during the arterial phase and washout during the portal venous or delayed phases, as outlined in guideline recommendations [10,25]. In instances where imaging alone was insufficient to confirm a diagnosis, a tumor biopsy followed by a histopathological analysis was conducted to ensure a thorough and accurate diagnostic process.

As part of the evaluation, liver function was assessed using the ALBI score (albumin–bilirubin), a tool designed to estimate the prognosis in hepatocellular carcinoma independently of the severity of the underlying liver fibrosis [26]. Additionally, staging was performed using the TNM system (tumor–node–metastasis) [27] and the BCLC (Barcelona Clinic Liver Cancer) classification [24].

### 2.3. Diagnosis of Cirrhosis and Comorbidities

In certain patients, the diagnosis of liver cirrhosis had been previously established based on the patient’s medical records. In newly identified cases, the diagnosis was initially suggested by clinical and ultrasound findings [28] and subsequently confirmed through liver elastography according to the elastography guidelines [29], or non-invasive biomarker panels such as FibroTest™ (BioPredictive, Paris, France) and FibroMax™ (BioPredictive, Paris, France). There are serum-based biochemical markers, with FibroMAX™ being a comprehensive panel commonly used in patients at risk for chronic liver disease. Within this panel, FibroTest™ quantitatively evaluates hepatic fibrosis [30].

The patients were evaluated for the presence of diabetes mellitus (DM) [31], hypertension (HTN) [32], chronic kidney disease (CKD) [33], and complex cardiac conditions (CC), including atrial fibrillation [34], ischemic cardiomyopathy [35], and heart failure [36]. The liver cirrhosis severity was assessed using the MELD (Model for End-Stage Liver Disease) score, which is based on serum bilirubin, creatinine, and INR. The Child–Pugh score was also utilized to evaluate liver function by incorporating ascites, hepatic encephalopathy, bilirubin, albumin, and INR [37].

### 2.4. Statistical Analysis

A data analysis was conducted using MedCalc Version 19.4 (MedCalc Software Corp., Brunswick, ME, USA) and Microsoft Office Excel 2019 (Microsoft for Windows). Descriptive statistics summarized the patients’ clinical data. The Kolmogorov–Smirnov test assessed the distribution of numerical variables. Variables with a normal distribution were reported as the mean ± standard deviation (SD), while those with a non-normal distribution were reported as the median with the interquartile range (IQR).

Categorical variables were expressed as frequencies and percentages. Group comparisons were performed using the Kruskal–Wallis H test, followed by a post-hoc analysis with the Mann–Whitney U test and Bonferroni correction for multiple comparisons. The Student’s t-test was utilized for continuous variables when the distribution was normal, while the Mann–Whitney U test was applied to non-normal distributions. Univariate and multivariate regression analyses were conducted to identify independent predictors of mortality.

A survival analysis was performed using the Kaplan–Meier method to estimate survival probabilities and compare survival curves between groups. The log-rank test was used to assess statistical differences between the curves. Additionally, a Cox proportional hazards regression analysis was conducted to evaluate the association between independent variables and survival outcomes while adjusting for potential confounders. Statistical significance was set at a *p*-value of <0.05.

## 3. Results

The study was conducted over seven years (2018–2024), spanning 69 months and including 216 patients, of whom 72.2% were male, aged between 32 and 93 years, with a mean age of 67.31 ± 8.73 years. All patients were diagnosed with HCC based on clinical, biological, and imaging criteria.

The subjects were divided into three subgroups based on the timing of the diagnosis related to the COVID-19 pandemic. Patients diagnosed between 1 May 2018 and 31 March 2020 were included in the PreP group (*n* = 94), those diagnosed between 1 April 2020 and 28 February 2022 were included in the PandP group (*n* = 58), and those diagnosed between 1 March 2022 and 31 January 2024 were included in the PostP group (*n* = 64). The median follow-up times in the PreP, PandP, and PostP periods were 157.5 [1–2316], 159.5 [1–1586], and 183.5 [3–1147] days, respectively, with no statistically significant differences between them. The median survival times in the PreP, PandP, and PostP periods were 39 [1–244], 58 [1–248], and 52 [3–220] days, respectively, with no statistically significant differences between them (*p* > 0.05). The baseline characteristics, demographic data, and laboratory parameters of the included subjects are summarized in Table 1.

A series of laboratory parameters were compared among the three groups. The number of platelets was significantly higher in patients diagnosed post-pandemic than in those diagnosed before (*p* = 0.0280) or during the pandemic (*p* = 0.0061). The ALT values were significantly lower in the subjects diagnosed PostP compared to those diagnosed PreP (*p* = 0.0249). In contrast, the serum albumin and serum sodium values were higher in the subjects diagnosed PostP than in those diagnosed PreP (*p* = 0.0020 and *p* = 0.0144, respectively). No other significant differences were found regarding the laboratory tests in the three groups (Table 1).

**Figure 1:**
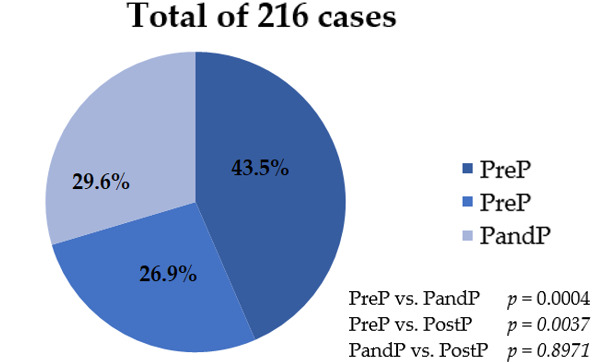
The number of patients diagnosed during the pandemic-related periods.

During the PandP and PostP periods, the numbers of newly diagnosed HCC cases decreased to 58 (26.9%) (*p* = 0.0004) and 64 (29.6%) (*p* = 0.0037), representing reductions of 38.3% and 31.9%, respectively, compared to the Prep period, which had 94 cases (43.4%) out of a total of 216 cases across all three periods (Figure 1).

Additionally, we analyzed the patient’s environment, diagnostic circumstances, presence of underlying liver disease, and stages of liver disease and HCC (Table 1).

The patients primarily originated from urban environments across all three groups (*p* = 0.0129, 0.0016, 0.0002) and were diagnosed either incidentally or during emergencies (*p* < 0.0001).

In all three groups, most patients had liver cirrhosis as an underlying condition (*p* < 0.0001), with a significant proportion being newly diagnosed concurrently with the diagnosis of HCC, without differences among the groups. Regarding the underlying condition, there were no differences among the three groups based on the Child–Pugh classification, nor within the same group (all *p* > 0.05) (Table 1).

**Figure 2:**
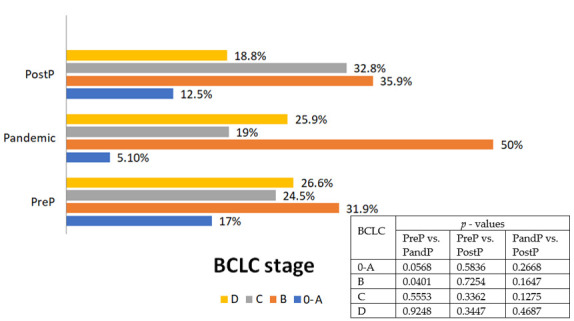
BCLC classification according to the year of the diagnosis.

No major differences were found among the three groups regarding HCC extension, including the number of lesions or TNM staging. However, a statistically significant distinction was observed in BCLC classification (*p* = 0.0401) between the PreP and PandP groups, where there was a higher proportion of patients in stage B and a decrease in stages 0–A in the PandP group. Most patients were categorized as BCLC stage B, stage D in the PreP and pandemic groups, and stage C in the PostP group (Figure 2).

The presence of portal vein thrombosis was observed in similar proportions of subjects across all three groups, predominantly malignant thrombosis (*p* < 0.0001) (Table 1).

As suggested by the TNM classification, which indicates that most patients had progressed beyond the early stages suitable for curative treatments, the proportion of patients undergoing surgical intervention was lowest in the pandemic and PostP groups (10.3% and 9.4%, respectively), followed by the PreP group (10.6%), with no statistically significant differences between groups (*p* = 0.8307, 0.9815, 0.8905).

The proportions of patients receiving percutaneous ablation were also similar across groups (11.7% in PreP, 8.6% in the pandemic group, and 7.8% in PostP), with no significant differences observed (*p* = 0.7391, 0.5966, 0.8652). In the PreP group, the proportion of patients receiving TACE (8.5%) was lower than that of those receiving percutaneous ablation (11.7%), although the differences between groups were not statistically significant (*p* = 0.2865, 0.3935, 0.9698). Across all three groups, the largest proportions of patients received systemic therapy, with 41.5% in PreP, 39.7% in the pandemic group, and 50% in PostP, although no significant differences were found (*p* = 0.9605, 0.3727, 0.3372). Similarly, best supportive care (BSC) was administered to 27.7% of patients in PreP, 25.9% in the pandemic group, and 18.8% in PostP, with no significant differences (*p* = 0.9563, 0.2737, 0.4687) (Table 2).

**Figure 3:**
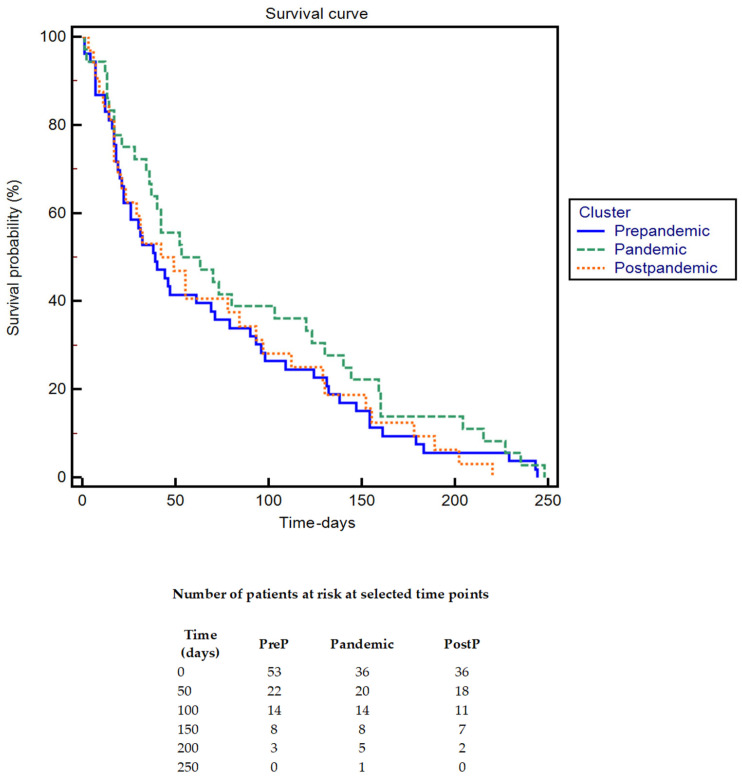
Kaplan–Meier survival curve.

The patients were followed for a duration of 9 months, during which a survival analysis was conducted. The Kaplan–Meier survival curve is displayed in Figure 3. No statistically significant differences in survival were observed between the groups (*p* > 0.05).

Table 3 summarizes the nine-month mortality rates based on BCLC stage. No significant differences were observed.

A univariate regression analysis was conducted, revealing the parameters associated with higher mortality rates: age (*p* = 0.036), AFP values (*p* = 0.0034), ALD etiology (*p* = 0.04), Child–Pugh stage (*p* = 0.0036), BCLC stage (*p* = 0.0003), TNM stage (*p* = 0.0039), malignant PVT (*p* = 0.0009), the presence of comorbidities (*p* = 0.001). The multivariate regression analysis evaluated the independent factors linked to increased mortality rates. The model, which included the presence of comorbidities (β = 0.044 ± 0.02, *p* < 0.0001, OR = 1.3 [1.07–1.51]), the BCLC stage (β = 0.038 ± 0.006, *p* < 0.0001, OR = 1.42 [1.10–1.66]), and malignant PVT (β = 0.023 ± 0.01, *p* < 0.0001, OR = 1.6 [1.41–1.83]), was linked to higher mortality rates. The predictors of mortality are summarized in Table 4.

## 4. Discussion

Our study investigated the impact of the COVID-19 pandemic on the evolution of patients with HCC by comparing the PreP, PandP, and PostP trends in diagnosed cases, treatments, and survival rates. During the pandemic, we observed a decline in newly diagnosed HCC cases. Although the number of diagnoses increased PostP, it did not return to PreP levels. Moreover, PandP was associated with a worsening of the BCLC stage of HCC due to a decrease in early BCLC A-0 cases. Our study found no significant differences in treatment characteristics or 9-month survival rates across the three periods.

The decline in diagnosed HCC cases during the PandP stage mirrors global trends reported in multiple studies [17,18,19,20,21,22,38], although its magnitude varies due to differences in regional pandemic timelines. The contributing factors include reduced healthcare access, resource reallocation [39], mobility restrictions, and fear of exposure [3]. In our cohort, the HCC diagnoses decreased by 31.91% in the PostP period compared to PreP (from 94 to 64 cases). Similarly, a U.S. study reported a 13% decline, although its findings may have been limited by the predominantly male veteran population [15]. Unlike other regions, Romania lacks a national HCC screening program [40], possibly explaining the slower diagnostic recovery post-pandemic.

Despite a slight increase in HCC cases during the PostP period, the overall downward trend in diagnoses continued. This may reflect patients’ reluctance to seek hospital care and increased COVID-19-related mortality among patients with chronic liver disease, particularly those with decompensated cirrhosis, who are at the highest risk for HCC [41,42].

Our study also revealed significant geographic disparities, with a growing predominance of urban cases across all periods (*p* = 0.0016, *p* = 0.0002), despite Romania’s nearly equal urban–rural population distribution. As Singal et al. highlighted, barriers such as income inequality, travel expenses, and limited access to specialized care may contribute to lower diagnosis rates and reduced access to curative treatment in rural populations [43].

Regarding the tumor characteristics, we observed a significant increase in the rate of BCLC-B cases during the PandP period. The proportion of patients in the BCLC-B stage increased from 31.9% in the PreP period to 50% during the PandP period (*p* < 0.0401). This trend was further emphasized by the higher number of patients with three lesions in the PandP period compared to the PreP period (2.1% vs. 10.4%, *p* = 0.0641). These findings highlight the pandemic’s impact on HCC diagnosis, leading to a worsening of the BCLC staging.

Although not statistically significant, we observed a decline in BCLC stage A diagnoses during the PandP period, from 17% to 5.2%, consistent with reports by Liang et al. [19] and Geh et al. [16], which attributed fewer early-stage detections to disrupted screening. Several studies also noted an increase in tumor size during PandP; one reported a rise from 48 ± 2.6 mm to 60 ± 4.6 mm (*p* = 0.017) [16], and another from Turkey showed an increase from 4.58 ± 3.77 cm to 7.42 ± 6.88 cm (*p* < 0.05) [20]. In contrast, larger studies found no significant differences in tumor size or stage [18,21,38], while a U.S. cohort reported more advanced T-stage diagnoses during the pandemic [19]. These discrepancies likely reflect regional differences in healthcare access and policy responses.

The COVID-19 pandemic initially disrupted HCC management processes; however, standardized protocols were rapidly implemented to ensure continuity of care [39]. Regional differences emerged, whereby studies from the UK and Japan reported reduced surgical wait times due to streamlined workflows [16,21], and the APASL guidelines recommended prioritizing locoregional therapies over surgery [44]. In Europe, a non-significant increase in TACE use was observed [38]. Conversely, prolonged treatment delays were noted in other regions, with a French study reporting an additional one-month delay and a UK–German cohort extending beyond the recommended timelines [45,46].

In our study, the treatment modalities showed no significant differences across the three periods, although specific trends were observed. For example, the TACE usage increased during the PandP period compared to the PreP period, which is consistent with the findings from other studies [38]. Surgical treatment remained relatively stable in its proportions between the PreP and PandP periods (10.6% vs. 10.3%), although the absolute number of surgeries dropped significantly during the PandP period (10 vs. 6 cases). This trend continued into the PostP period, where the proportion and number of surgical cases were similar to those in the PandP period (9.4%, 6 cases).

Although the trend observed during the PostP period did not achieve statistical significance, there was an increase in the proportion of patients receiving TACE, which reached 50%, the highest among the three evaluated periods. This increase was accompanied by a decrease in the proportion of patients classified for BSC, dropping from 27.7% in the PreP period to 25.9% in the PandP period and 18.8% in the PostP period. Meanwhile, the proportions of other therapeutic options remained relatively stable. A possible explanation for this trend is the reduction in BCLC D cases during the PostP period, suggesting a shift toward less advanced liver pathologies. This is further supported by improvements in platelet count, ALT, albumin, and serum sodium levels when comparing the PreP and PostP periods.

Our study found no significant differences in survival across the three periods (*p* > 0.05) in the Kaplan–Meier 9-month survival analysis. We analyzed nine-month mortality rates according to the BCLC stage, observing no significant differences across the three periods. Our findings align with previous studies that reported stable survival rates despite variations in treatment approaches [19] or delays in care [46]. However, it is important to note that these studies assessed survival over slightly longer timeframes—one year [46] and 11 months [19]—compared to our nine-month analysis. Since HCC is generally a slow-growing tumor, detecting significant survival differences over shorter observation periods can be challenging [47].

The Cox regression analysis showed that in univariate models, higher mortality was associated with age, AFP levels, ALD etiology, Child–Pugh and BCLC stages, TNM stage, malignant portal vein thrombosis (PVT), and comorbidities. The multivariate analysis identified comorbidities, the BCLC stage, and malignant PVT as independent predictors. These findings underscore the importance of an early diagnosis through structured screening programs, which were disrupted during the pandemic and have yet to fully recover.

Several limitations of this study should be acknowledged. First, although our center serves a large geographic region, the findings may not fully reflect the situation across Romania. The relatively small number of cases may explain the absence of significant differences in the treatment received and survival, highlighting the need for prospective multicenter studies to assess the impact of pandemic-related policies on HCC management during COVID-19. Second, the relatively short follow-up period of nine months limits the ability to assess the long-term survival outcomes. Future studies with extended follow-up durations are needed to better understand the pandemic’s long-term effects on HCC survival rates and associated factors.

Lastly, while the retrospective design of this study presents certain limitations, it also provides valuable insights into the decline in HCC diagnoses during the PandP and PostP periods. Although we were unable to assess additional contributing factors—such as variations in alcohol consumption, access to healthcare services, the reallocation of medical resources, social distancing measures, patients’ reluctance to visit hospitals, and the impacts of the median time from diagnosis to treatment on mortality and survival—these are recognized as important considerations for future research.

## 5. Conclusions

The COVID-19 pandemic led to a decline in HCC diagnoses, which increased during the post-pandemic period but did not return to pre-pandemic levels. During the pandemic, there was a worsening of BCLC grades and an increase in the number of tumors compared to the pre-pandemic period. Despite this, we did not observe significant changes in BCLC classification, TNM staging, or treatment approaches in the post-pandemic period compared to the pre-pandemic period—indicating signs of recovery in HCC diagnosis and a gradual return to pre-pandemic conditions. Similarly, the nine-month survival rates showed no significant differences across the three evaluated periods. Our study highlights the need for further research on the pandemic’s long-term impact on HCC management, considering geographic and socioeconomic factors in HCC diagnosis and treatment.

## Figures and Tables

**Table 1:** Patient characteristics and HCC staging.

Parameter	Overall (*n* = 216)	I. PreP Group (*n* = 94)	II. PandP Group (*n* = 58)	III. PostP Group (*n* = 64)	*p* Values		
					I/II	I/III	II/III
Mean age (years)	67.3 ± 8.7	67 ± 8.8	66 ± 9.6	68.9 ± 7.7	0.5120	0.1634	0.2283
Gender (%)							
Females	60 (27.8%)	(26) 27.7%	14 (24.1%)	20 (31.3%)	0.7642	0.7562	0.4943
Males	156 (72.2%)	(68) 72.3%	44 (75.9%)	44 (68.7%)	0.7642	0.7562	0.4943
Etiology (%)							
ALD	45 (20.8%)	18 (19.1%)	13 (22.4%)	14 (21.9%)	0.7768	0.8194	0.8793
HBV	37 (17.1%)	17 (18.1%)	7 (12.1%)	13 (20.3%)	0.4496	0.8890	0.3287
HBV + HCV	5 (2.3%)	2 (2.1%)	2 (3.5%)	1 (1.6%)	0.9982	0.7130	0.9278
HBV + HDV	12 (5.6%)	6 (6.4%)	3 (5.2%)	3 (4.7%)	0.9608	0.9181	0.7710
HCV	88 (40.8%)	39 (41.5%)	23 (39.7%)	26 (40.6%)	0.9605	0.9587	0.9333
MASLD	29 (13.4%)	12 (12.8%)	10 (17.2%)	7 (10.9%)	0.6089	0.9114	0.4577
Performance index	1 [0–3]	1 [0–3]	1 [0–3]	1 [0–3]	0.7474	0.8544	0.5517
Platelet count (×10^3^/µL)	175 [33–715]	142.5 [33–388]	142.5 [42–438]	186 [60–715]	0.6904	0.0280	0.0061
ALT(UI/L)	45 [8–816]	49.5 [10–566]	44.5 [10–685]	38 [8–816]	0.1367	0.0249	0.3848
ALB (mg/dL)	2.9 [1.2–5.2]	2.75 [1.2–4.7]	2.75 [1.6–4.5]	3.15 [1.2–5.2]	0.3229	0.0020	0.0507
AFP (ng/mL)	73 [0.1–191,000]	63.1 [1.5–191,000]	215.9 [0.1–99,500]	63.5 [0.1–29,800]	0.2822	0.5649	0.1632
Serum sodium (mg/dL)	138 [114–147]	137 [124–144]	138 [126–146]	138 [114–147]	0.0612	0.0144	0.4956
Environment:							
Rural	79 (36.6%)	38 (40.4%)	20 (34.5%)	21 (32.8%)	0.5785	0.4225	0.9947
Urban	137 (63.4%)	56 (59.6%)	38 (65.5%)	43 (67.2%)	0.5785	0.4225	0.9947
Cirrhosis (%)							
Yes	200 (92.6%)	91 (96.8%)	51 (87.9%)	58 (90.6%)	0.0701	0.1938	0.8503
No	16 (7.4%)	3 (3.2%)	7 (12.1%)	6 (9.4%)	0.0701	0.1938	0.8503
Newly diagnosed cirrhosis							
Yes	90 (45%)	40 (44%)	24 (47.1%)	26 (44.8%)	0.8568	0.9416	0. 9618
No	110 (55%)	51 (56%)	27 (52.9%)	32 (55.2%)	0.8568	0.9416	0.9618
Child-Pugh grade (%)							
A [5–6]	66 (30.6%)	28 (29.8%)	17 (29.3%)	21 (32.8%)	0.9066	0.8220	0.8250
B [7–9]	81 (37.5%)	37 (39.4%)	19 (32.8%)	25 (39.1%)	0.5181	0.8981	0.5928
C [10–15]	69 (31.9%)	29 (30.8%)	22 (37.9%)	18 (28.2%)	0.4691	0.8621	0.3439
MELD score	18 [11–33]	19 [15–33]	17 [13–26]	18 [11–30]	0.2235	0.4587	0.8480
Comorbidities							
CC	52 (24.1%)	21 (22.3%)	12 (20.7%)	19 (29.7%)	0.9761	0.3876	0.3514
HTN	93 (43.1%)	39 (41.5%)	20 (34.5%)	34 (53.1%)	0.4909	0.2029	0.0597
CKD	10 (4.6%)	7 (7.4%)	1 (1.7%)	2 (3.1%)	0.2465	0.4250	0.9306
DM	72 (33.4%)	38 (40.4%)	15 (25.9%)	19 (29.7%)	0.995	0.2277	0.7908
ALBI score	−1.81 ± 0.47	−1.83 ± 0.38	−1.81 ± 0.55	−1.76 ± 0.57	0.7915	0.3556	0.6236
Number of HCC lesions:							
1 lesion	100 (46.3%)	47 (50%)	22 (37.9%)	31 (48.5%)	0.1978	0.9816	0.3189
2 lesions	20 (9.3%)	8 (8.5%)	5 (8.6%)	7 (10.9%)	0.7815	0.8189	0.9031
3 lesions	10 (4.6%)	2 (2.1%)	6 (10.4%)	2 (3.1%)	0.0641	0.9016	0.2079
Infiltrative (diffuse)	16 (7.4%)	8 (8.5%)	3 (5.2%)	5 (7.8%)	0.6596	0.8904	0.8313
Multiple lesions	70 (32.4%)	29 (30.9%)	22 (37.9%)	19(29.7%)	0.4771	0.9879	0.449
Tumor size (cm):							
Single lesion	5.5 ± 2.1	5.8 ± 3.1	4.8 ± 1.8	5.6 ± 1.9	0.8044	0.6280	0.7250
Infiltrative/Multiple lesions	6.1 ± 2.9	5.9 ± 1.8	6.1 ± 1.6	6.5 ± 2.8	0.6311	0.4562	0.5419
TNM							
IA	12 (5.6%)	9 (9.6%)	1 (1.7%)	2 (3.1%)	0.1162	0.2087	0.9306
IB	58 (26.9%)	21 (21.3%)	17 (29.3%)	20 31.3%)	0.3566	0.2179	0.9159
II	37 (17.1%)	20 (22.3%)	9 (15.5%)	8 (12.5%)	0.4197	0.1759	0.8289
IIIA	38 (17.6%)	17 (18.1%)	11 (19%)	10 (15.6%)	0.9392	0.8457	0.7974
IIIB	45 (20.8%)	16 (17%)	13 (22.5%)	16 (25%)	0.5316	0.3044	0.9117
IVA	7 (3.2%)	5 (5.3%)	1 (1.7%)	1 (1.6%)	0.4963	0.4412	0.5036
IVB	19 (8.8%)	6 (6.4%)	6 (10.3%)	7 (10.9%)	0.5777	0.4741	0.8518
HCC stage (BCLC) (%)							
0-A	27 (12.5%)	16 (17%)	3 (5.2%)	8 (12.5%)	0.0595	0.5836	0.2763
B	82 (38%)	30 (31.9%)	29 (50%)	23 (35.9%)	0.0401	0.7254	0.1647
C	55 (25.5%)	23 (24.5%)	11 (19%)	21 (32.8%)	0.5553	0.3362	0.1275
D	52(24%)	25 (26.6%)	15 (25.8%)	12 (18.8%)	0.9356	0.3447	0.4766
PVT (%)	75 (34.7%)	31 (33%)	19 (32.8%)	25 (39%)	0.8791	0.5454	0.6006
Benign	7/75 (8%)	3 (9.7%)	2 (10.5%)	1 (4%)	0.6936	0.7632	0.6852
Malignan	69/75 (92%)	90.3% (28)	17 (89.5%)	24 (96%)	0.6936	0.7632	0.6852

AFP—alpha-fetoprotein; ALB—total serum albumin levels; ALBI—albumin–bilirubin score; ALD—alcohol-related liver disease; ALT—alanine aminotransferase; CC—complex cardiac disease; CKD—chronic kidney disease; DM—diabetes mellitus; HBV—hepatitis B virus; HCC—hepatocellular carcinoma; HCV—hepatitis C virus; HDV—hepatitis D virus; HTN—arterial hypertension; MASLD—metabolic dysfunction–associated steatotic liver disease; MELD—Model for End-Stage Liver Disease; n—number; PVT—portal vein thrombosis; TNM—tumor, node, metastasis staging system.

**Table 2:** Treatment characteristics.

Treatment	Overall (*n* = 216)	PreP Group (*n* = 94)	Pandemic Group (*n* = 58)	PostP Group (*n* = 64)	*p* Values		
					I/II	I/III	II/III
Systemic	94 (43.5%)	39 (41.5%)	23 (39.7%)	32 (50%)	0.9605	0.3727	0.3372
Surgery	23 (10.7%)	10 (10.6%)	6 (10.3%)	6 (9.4%)	0.8307	0.9815	0.8905
Percutaneous ablation	20 (9.3%)	11 (11.7%)	5 (8.6%)	5 (7.8%)	0.7391	0.5966	0.8652
TACE	26 (12%)	8 (8.5%)	9 (15.5%)	9 (14.1%)	0.2865	0.3935	0.9698
BSC	53 (24.5%)	26 (27.7%)	15 (25.9%)	12 (18.8%)	0.9563	0.2737	0.4687
BCLC classification according to treatment							
BCLC stage		Systemic (*n* = 94)	Surgery (*n* = 23)	Percutaneous ablation (*n* = 20)	TACE (*n* = 26)	BSC (*n* = 53)	
	A	2 (2.1%)	7 (30.4%)	14 (70%)	3 (11.6%)	1 (1.9%)	
	B	41 (43.6%)	16 (69.6%)	2 (10%)	23 (88.5%)	0%	
	C	51 (54.3%)	0%	4 (20%)	0%	0%	
	D	0%	0%	0%	0%	52 (98.1%)	

TACE—transarterial chemoembolization; BSC—best supportive care.

**Table 3:** Nine-month mortality rates according to BCLC stage.

BCLC Stage	I.PreP Group (*n* = 94)	II.Pandemic Group (*n* = 58)	III.PostP Group (*n* = 64)	*p* Values		
				I/II	I/III	II/III
0-A	8/16 (50%)	2/3 (66.7%)	2/8 (25%)	0.9216	0.4642	0.5641
B	16/30 (53.4%)	17/29 (58.6%)	8/23 (34.8%)	0.8887	0.2847	0.1537
C	14/23 (60.9%)	6/11 (54.5%)	8/21 (33.4%)	0.9859	0.1280	0.4382
D	16/25 (64%)	11/15 (73.4%)	10/12 (84.4%)	0.7903	0.3736	0.8265

BCLC—Barcelona Clinic Liver Cancer Staging System.

**Table 4:** Predictors of mortality.

Baseline Parameters	Univariate Analysis		Multivariate Analysis	
	HR (95% CI)	*p*-Value	HR (95% CI)	*p*-Value
Age	1.303 (1.112–2.289)	0.036		
AFP values	1.601 (1.332–1.835)	0.0034		
ALD etiology	1.466 (1.679–2.815)	0.04		
Child-Pugh stage	1.704 (1.441–2.924)	0.0036		
BCLC stage	1.663 (1.032–2.406)	0.0003	1.363 (1.032–2.406)	<0.0001
TNM stage	1.215 (1.002–2.342)	0.0039		
Malignant PVT	2.16 (1.902–3.974)	0.0009	1.816 (1.330–2.724)	<0.0001
Presence of comorbidities	1.983 (1.421–3.268)	0.001	1.623 (1.223–2.681)	<0.0001

AFP—alpha-fetoprotein; ALD—alcohol-related liver disease; BCLC—Barcelona Clinic Liver Cancer Staging System; PVT—portal vein thrombosis; TNM—tumor, node, metastasis staging system. A Cox regression analysis was conducted to identify factors associated with mortality during admission, utilizing a significance level of 0.05.

## Data Availability

Data are available upon request.
